# Mammography-based artificial intelligence for breast cancer detection, diagnosis, and BI-RADS categorization using multi-view and multi-level convolutional neural networks

**DOI:** 10.1186/s13244-025-01983-x

**Published:** 2025-05-21

**Authors:** Hongna Tan, Qingxia Wu, Yaping Wu, Bingjie Zheng, Bo Wang, Yan Chen, Lijuan Du, Jing Zhou, Fangfang Fu, Huihui Guo, Cong Fu, Lun Ma, Pei Dong, Zhong Xue, Dinggang Shen, Meiyun Wang

**Affiliations:** 1 Department of Radiology, Henan Provincial People’s Hospital & People’s Hospital of Zhengzhou University, Zhengzhou, China; 2 Imaging Diagnosis of Neurological Diseases and Research Laboratory of Henan Province, Zhengzhou, China; 3 Beijing United Imaging Research Institute of Intelligent Imaging, Beijing, China; 4 United Imaging Intelligence (Beijing) Co. Ltd., Beijing, China; 5 Department of Radiology, Henan Cancer Hospital, Affiliated Cancer Hospital of Zhengzhou University Zhengzhou, Henan, China; 6 Department of Radiology, The First Affiliated Hospital of Zhengzhou University, Zhengzhou, China; 7 Department of Radiology, The Third Affiliated Hospital of Zhengzhou University, Zhengzhou, China; 8 Department of Radiology, Zhengzhou Central Hospital, Zhengzhou, China; 9 Department of Radiology, Fuwai Central China Cardiovascular Hospital, Zhengzhou, China; 10 Shanghai United Imaging Intelligence Co. Ltd, Shanghai, China; 11 School of Biomedical Engineering, ShanghaiTech University, Shanghai, China

**Keywords:** Artificial intelligence, Breast neoplasms, Deep learning, Diagnosis, Mammography

## Abstract

**Purpose:**

We developed an artificial intelligence system (AIS) using multi-view multi-level convolutional neural networks for breast cancer detection, diagnosis, and BI-RADS categorization support in mammography.

**Methods:**

Twenty-four thousand eight hundred sixty-six breasts from 12,433 Asian women between August 2012 and December 2018 were enrolled. The study consisted of three parts: (1) evaluation of AIS performance in malignancy diagnosis; (2) stratified analysis of BI-RADS 3–4 subgroups with AIS; and (3) reassessment of BI-RADS 0 breasts with AIS assistance. We further evaluate AIS by conducting a counterbalance-designed AI-assisted study, where ten radiologists read 1302 cases with/without AIS assistance. The area under the receiver operating characteristic curve (AUC), sensitivity, specificity, accuracy, and F1 score were measured.

**Results:**

The AIS yielded AUC values of 0.995, 0.933, and 0.947 for malignancy diagnosis in the validation set, testing set 1, and testing set 2, respectively. Within BI-RADS 3–4 subgroups with pathological results, AIS downgraded 83.1% of false-positives into benign groups, and upgraded 54.1% of false-negatives into malignant groups. AIS also successfully assisted radiologists in identifying 7 out of 43 malignancies initially diagnosed with BI-RADS 0, with a specificity of 96.7%. In the counterbalance-designed AI-assisted study, the average AUC across ten readers significantly improved with AIS assistance (*p* = 0.001).

**Conclusion:**

AIS can accurately detect and diagnose breast cancer on mammography and further serve as a supportive tool for BI-RADS categorization.

**Critical relevance statement:**

An AI risk assessment tool employing deep learning algorithms was developed and validated for enhancing breast cancer diagnosis from mammograms, to improve risk stratification accuracy, particularly in patients with dense breasts, and serve as a decision support aid for radiologists.

**Key Points:**

- The false positive and negative rates of mammography diagnosis remain high.
- The AIS can yield a high AUC for malignancy diagnosis.
- The AIS is important in stratifying BI-RADS categorization.

**Graphical Abstract:**

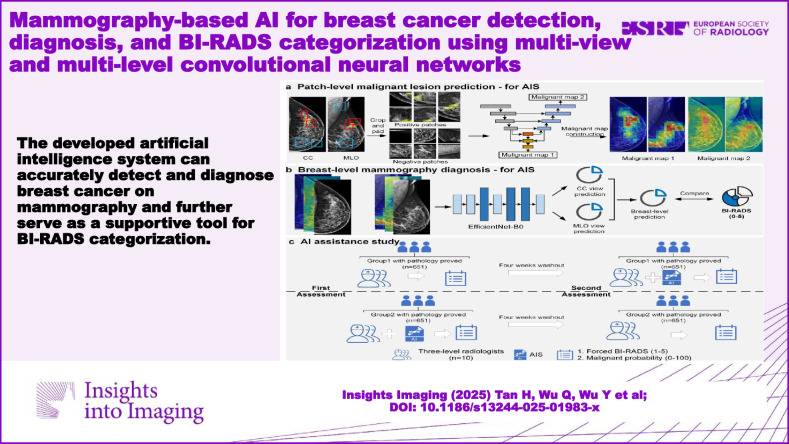

## Introduction

Breast cancer is the leading cause of tumor-related deaths among women, and its incidence rate is increasing [1]. Mammography is one of the most common imaging modalities for breast cancer screening, and early diagnosis plays an important role in reducing mortality rates [2–4]. In routine clinical workflows, the Breast Imaging Reporting and Data System (BI-RADS) published by the American College of Radiology provides standardized guidelines for mammographic reports and facilitates breast cancer diagnosis and management [5, 6]. However, the false positive and false negative rates of human manual mammographic interpretation remain high owing to individual differences (particularly breast density) and the lack of experienced radiologists [7–10]. Typically, incorrect readings classified as BI-RADS 0 or 4 categories often lead to additional imaging or unnecessary biopsies [11, 12]. For example, false-positive results (based on pathological reports) for BI-RADS 4 category could be due to the wide range of malignant prediction (2–95%), limiting its clinical application for individual diagnosis. Therefore, there is a need to establish a clinically applicable breast cancer diagnosis method for assisting decision-making on BI-RADS categorization.

Recent advances in deep learning-based artificial intelligence (AI) methods [13–17] have exhibited great potential in breast cancer detection and diagnosis. Some works [18–21] demonstrated that AI could match or surpass human interpretation in mammography. However, some of the potential roles of AI have yet to be fully addressed. Many studies assessed mammography using forced BI-RADS categorization (only 1–5) [18, 19, 22] and focused on comparing the performance of current clinical criteria and BI-RADS lexicon with those of AI. However, further research is needed on whether AI can serve as a supportive tool to improve clinical decision-making on routine BI-RADS categorization, e.g., easier interpretation of BI-RADS 0 and better classification of BI-RADS 3 and 4 groups. Beyond that, previous large-scale AI-based mammographic studies have paid little attention to Asian women due to their high proportion of dense parenchyma and blurred lesions on mammograms. Therefore, the potential application of AI in detecting and diagnosing lesions in dense parenchyma, and obscuring lesions, also needs further investigation.

To move beyond the limitations of previous AI approaches and accelerate broader adoption of deep-learning techniques by clinicians, we developed a two-step AI system (AIS) that utilizes multi-view mammograms and multi-level mammographic features to automatically detect and diagnose breast cancer in the Asian population. We then explored the benefits of AIS on assisted decision-making for BI-RADS 0, 3, and 4 categories by conducting a stratified analysis and counterbalance-designed reader study.

## Material and methods

### Study sample and outcomes

This retrospective multi-cohort study was approved by the Institutional Review Board of Clinic 1 and Clinic 2. De-identified data were collected with a waiver of written informed consent.

**Fig. 1:**
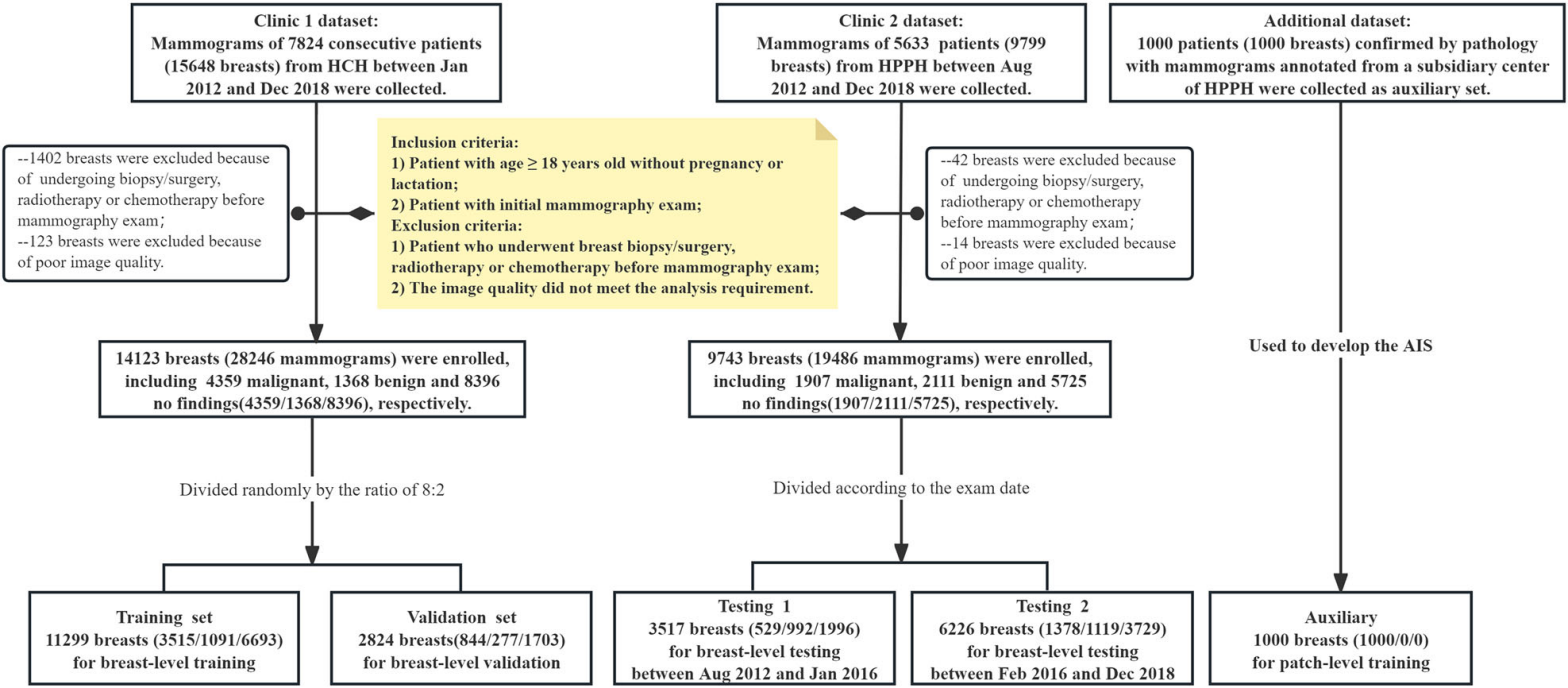
The workflow of study inclusion and exclusion

This study included both screening and diagnostic mammograms. The inclusion criteria were as follows: (1) patients underwent initial mammography examination; (2) patients with determinate mammography abnormality confirmed by surgery or biopsy pathology; and (3) patients without abnormal mammography findings had more than 2 years of normal imaging follow-up. The exclusion criteria were as follows: (1) patients with age no more than 18 years old, pregnant, or lactating women; (2) patients who had undergone biopsy/surgery, radiotherapy, chemotherapy, and other treatments before mammography examination; (3) patients with blurry image display or incomplete lesion package. A total of 12,433 women (24,866 breasts) were enrolled in our study according to the inclusion and exclusion criteria (Fig. 1). Here, the right and left breasts from one patient were considered as two samples. In this article, the word “breast” refers to the unilateral breast. Each breast had two paired views, i.e., CC and MLO images, which means one breast contains two mammograms. And all mammograms were acquired on the Hologic Selenia Dimensions digital mammography system (Hologic, Inc.) with a spacing of 0.07 mm and a resolution of 2560 × 3328–3328 × 4096.

Altogether, we collected 28,246 consecutive digital mammograms (14,123 breasts) from Clinic 1 between January 2015 and December 2018, and randomly split them by a ratio of 8:2 into training and validation sets to develop and validate AIS at the breast level. We also collected 7034 mammograms (3517 breasts) from Clinic 2 between August 2012 and January 2016 as testing set 1 and 12,452 consecutive mammograms (6226 breasts) from Clinic 2 between February 2016 and December 2018 as testing set 2 to test AIS at the breast level. Test Set 1 and Test Set 2 are both independent and external to the training data. To facilitate the training of AIS, another 2000 mammograms (1000 breasts confirmed by pathology) with annotation from a subsidiary center between January 2018 and December 2020 were collected as an auxiliary set, which were only used in patch-level training and validation. All detailed dataset information was shown in Table 1.

**Table 1:** Dataset

Dataset	Total	Malignant	Benign	No-findings	Age (mean (SD))	Patch-level	Breast-level	ROI
Training	11,299	3515	1091	6693	51.3 (10.5)		Train	×
Validation	2824	844	277	1703	51.2 (10.3)		Validation	×
Testing 1	3517	529	992	1996	46.4 (9.2)		Test	×
Testing 2	6226	1378	1119	3729	48.1 (10.0)		Test	×
Auxiliary	1000	1000				Train/validation		√

Data are a number of breasts, and are the total number of breasts used to train, validate, and test the AIS. Malignant and benign groups were confirmed by biopsy or surgical excision, and the no-finding group was defined as those without clinical findings for more than 2 years of follow-up imaging

*AIS* artificial intelligence system, *ROI* region of interest, *SD* standard deviation

### Development of AIS

**Fig. 2:**
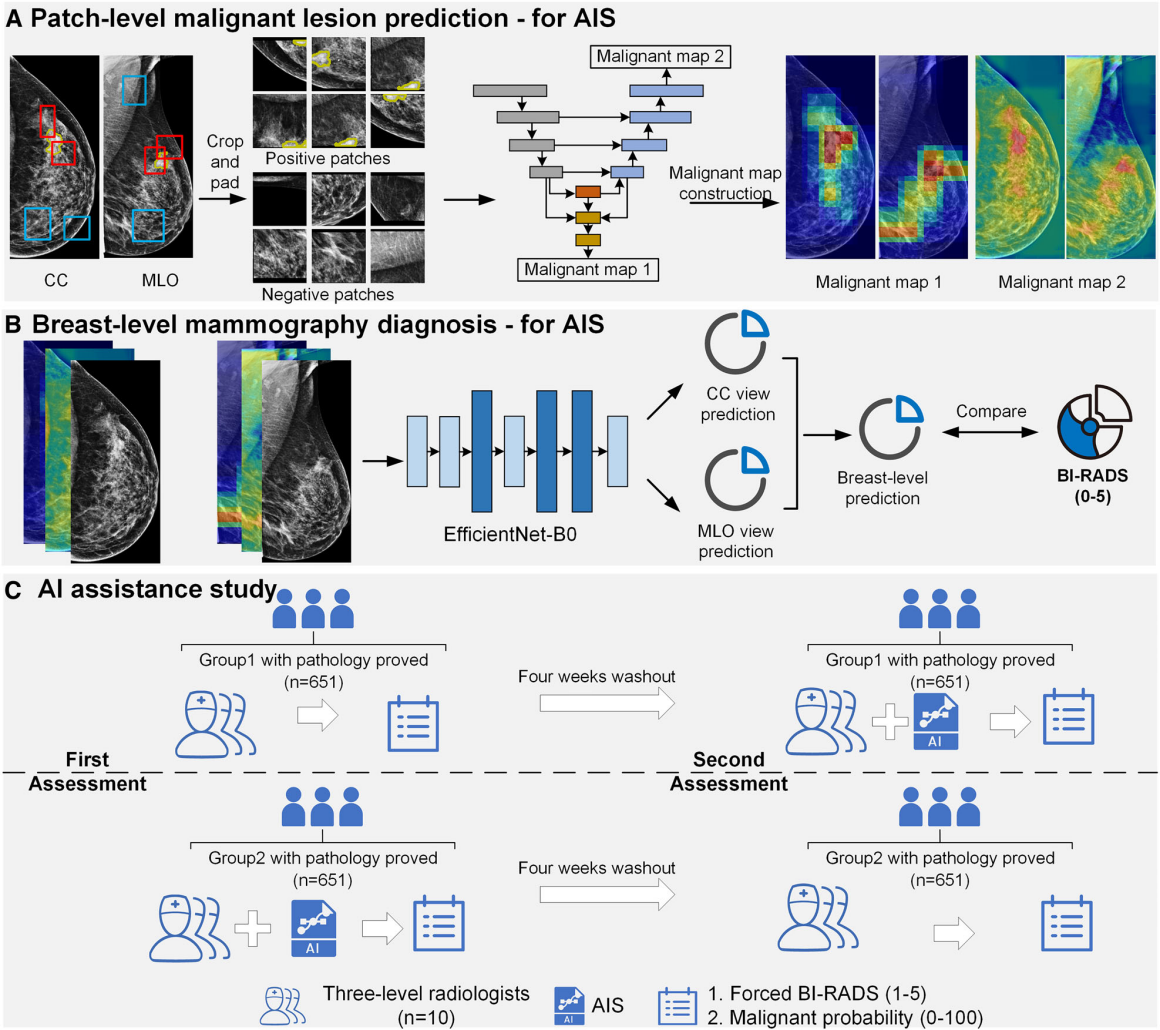
The workflow of the study. **A** The first step of the AIS. **B** The second step of the AIS. **C** The workflow of the AI assistance study. BI-RADS, breast imaging reporting and data system; AIS, artificial intelligence system

In this study, we proposed a multi-view multi-level AIS for breast cancer detection and diagnosis. It contains two steps: (1) patch-level malignant lesion localization (Fig. 2A); (2) breast-level malignancy diagnosis (Fig. 2B). In step one, a patch-level multi-task network was proposed to jointly learn suspicious lesions’ localization and characteristics (malignant or non-malignant). We cropped the original images into 512 × 512 patches randomly and fed them into the multi-task network to get patch-level tumors’ segmentation and classification. Then, we merged patch-level classification outputs into Malignant Map 1, which showed a coarse heat map of malignant lesion characteristics. Finally, we merged patch-level segmentation outputs into Malignant Map 2, which localized the suspicious malignant region. In step two, we used the original CC and MLO images together with Malignant Map 1 and 2 as a three-channel input to train an EfficientNet-B0 for image-level classification, aiming to learn the macroscopic malignancy features. The input images were re-sized to a resolution of 1100 × 600 pixels to fit the EfficientNet-B0 architecture. We utilized transfer learning by initializing the EfficientNet-B0 model with weights pre-trained on ImageNet. We averaged the EfficientNet-B0 prediction from the CC view and MLO view to get the breast-level cancer classification. Here, we referred to the breast-level predicted probability from EfficientNet-B0 as the AIS score, and a higher score indicated a higher probability of malignancy.

### Stratified analysis of BI-RADS 3–4 subgroups

Due to the high uncertainty for BI-RADS 3–4 categories (0–95% malignant likelihood), we conducted a stratified analysis to test the ability of AIS to up-/down-grade BI-RADS 3–4 findings. Two senior radiologists with more than 10 years’ experience provided a BI-RADS category (0–5) among breasts that had definitive pathological results (malignant or benign). For this stratified analysis, we included only those cases where both senior radiologists provided concordant BI-RADS assessments. Specifically, a breast was included in the analysis only when both radiologists independently assigned the same BI-RADS category. And the detailed BI-RADS category is shown on Supplementary Table S1. Specifically, a breast was assigned positive if it had a BI-RADS score of 4A, 4B, 4C, or 5, and as negative if it had a BI-RADS score of 1, 2, or 3. Herein, we used 4A as the threshold for malignancy, rather than 3, as 4A had a balanced sensitivity and specificity and yielded the highest accuracy for predicting malignancy using BI-RADS categorization. Breasts with a BI-RADS score of 0 were not included in this stratified analysis. For comparison with BI-RADS reports, AIS predicted the malignancy with a cutoff AIS score of 0.5.

### Re-assessment of BI-RADS 0 with AIS assistance

In clinical practice, BI-RADS 0 patients require further evaluation or recall for additional imaging, so we conducted a separate reassessment study for the BI-RADS 0 subgroup. The breasts that were initially evaluated as BI-RADS 0 category in the BI-RADS 3–4 stratified analysis were re-assessed by the same radiologists with AIS assistance after a more than four-week washout period. On the AI-aided interface, experts can see a red contour of the suspicious area and the probability of malignancy predicted by the AIS. Experts had access to the suspicious area immediately when loading mammograms, and the suspicious area could be toggled off to reveal the unaltered mammograms. Given the AIS results, experts had the option to take it into consideration or disregard it based on clinical judgment.

### AIS assistance study

We further conducted a reader study to assess the performance of 10 radiologists with three degrees of expertise (senior: breast imaging diagnosis experience ≥ 10 years, mid-level: breast imaging diagnosis experience 3–10 years, and junior: breast imaging diagnosis experience 0–3 years) responsible for mammography interpretation by asking each reader to provide a forced BI-RADS score (1–5) and a probability of malignancy score (0–100) with/without AIS assistance (Fig. 2C). Here, we used forced BI-RADS score (1–5) other than BI-RADS category (0–5) in the stratified analysis of BI-RADS 3–4 subgroups due to the uncertainty of BI-RADS 0 category.

The study was conducted among 1302 breasts randomly selected from the testing sets with pathological proven. Readers read 651 breasts without AIS and the other 651 breasts with AIS during the first session, and crossover during the second session, where a four-week washout period was separated. All readers were blinded to pathology results.

### Statistical analysis

Statistical analysis was performed with R, version 4.0.1 software (R Project for Statistical Computing). To evaluate the diagnostic ability of the AIS, we plotted the receiver operating characteristic curves (ROC) and assessed AUC, accuracy, sensitivity, specificity, and F1 score at the breast level. Intraclass correlation coefficient (ICC) was used to evaluate the agreement between radiologists with/without AIS. *p* < 0.05 indicated a statistically significant difference.

## Results

### Patient characteristics

A total of 24,866 breasts (mean age 49.8 years ± 10.3) were included, 10,745 (43.2%) were confirmed by biopsy or surgical pathology (7266 were malignancy and 3479 were benign), and 14,121 (56.8%) had no findings with more than 2 years of follow-up imaging (Table 1).

### Performance of AIS

**Fig. 3:**
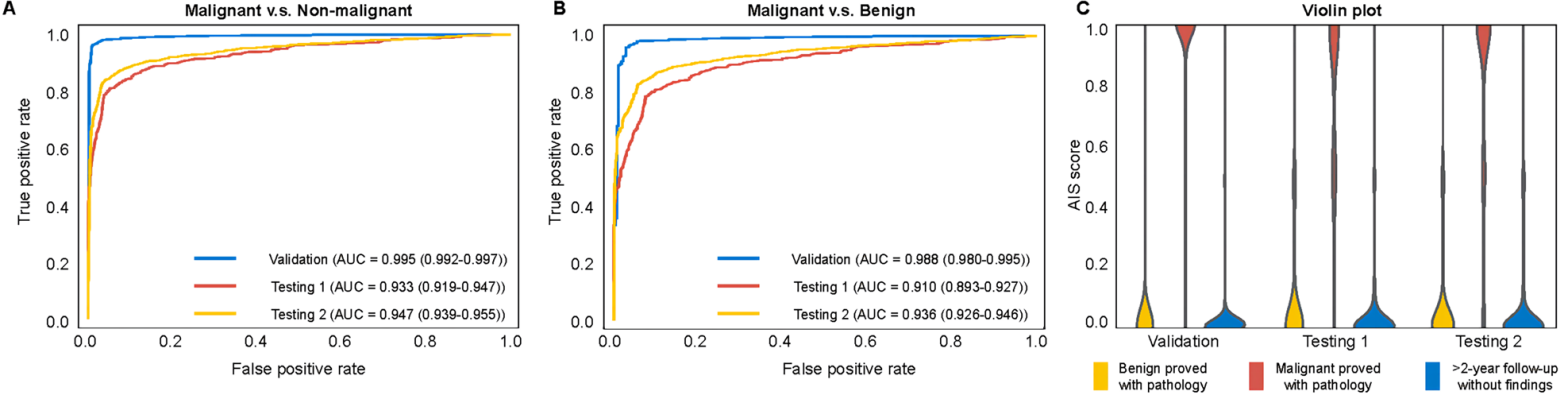
Stratified analysis of gland type and lesion type, and cancer type. **A** A stratified analysis of gland type. **B** A stratified analysis of lesion type. **C** A stratified analysis of cancer type

The performance of AIS is shown in Table 2 and Fig. 3. When discriminating malignant breasts from non-malignant breasts (benign group + no-finding group), AIS achieved an AUC of 0.995 (95% CI: 0.992–0.997), 0.933 (95% CI: 0.919–0.947), and 0.947 (95% CI: 0.939–0.955) in the validation set, testing set 1 and testing set 2, respectively. When discriminating malignant breasts from benign breasts, the AIS achieved an AUC of 0.988 (95% CI: 0.980–0.995), 0.910 (95% CI: 0.893–0.927), and 0.936 (95% CI: 0.926–0.946) in the validation set, testing set 1, and testing set 2, respectively.

**Table 2:** The performance of the AIS

Type	Dataset	AUC	Sensitivity (%)	Specificity (%)	Accuracy (%)	F1
Malignant vs non-malignant	Validation	0.995 (0.992, 0.997)	96.5 [814/844] (95.0, 97.6)	98.9 [1959/1980] (98.4, 99.3)	98.2 [2773/2824] (97.6, 98.7)	0.970
	Testing 1	0.933 (0.919, 0.947)	81.1 [429/529] (77.5, 84.3)	95.0 [2838/2988] (94.1, 95.7)	92.9 [3267/3517] (92.0, 93.7)	0.774
	Testing 2	0.947 (0.939, 0.955)	84.0 [1158/1378] (82.0, 85.9)	96.0 [4654/4848] (95.4, 96.5)	93.3 [5812/6226] (92.7, 94.0)	0.848
Malignant vs benign	Validation	0.988 (0.980, 0.995)	96.1 [811/844] (94.5, 97.3)	97.1 [269/277] (94.4, 98.8)	96.3 [1080/1121] (95.1, 97.4)	0.975
	Testing 1	0.910 (0.893, 0.927)	80.3 [425/529] (76.7, 83.6)	91.1 [904/992] (89.2, 92.8)	87.4 [1329/1521] (85.6, 89.0)	0.816
	Testing 2	0.936 (0.926, 0.946)	84.4 [1163/1378] (82.4, 86.3)	92.9 [1040/1119] (91.3, 94.4)	88.2 [2203/2497] (86.9, 89.5)	0.888

Numbers in brackets are the numerator/denominator, and numbers in parentheses are the 95% CI

*AUC* area under the receiver operating characteristic curve, *CI* confidence interval, *AIS* artificial intelligence system

### The benefit of AIS for stratifying BI-RADS 3–4 subgroups

**Fig. 4:**
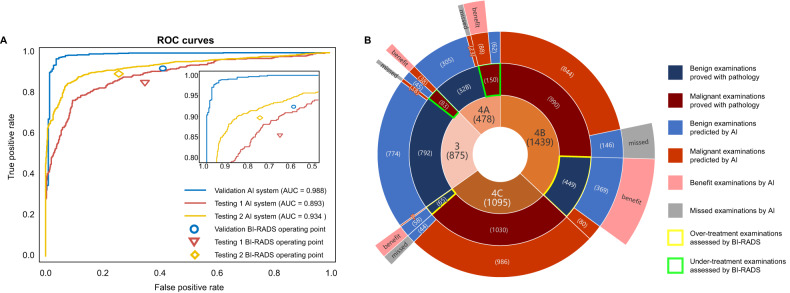
Stratified analysis of BI-RADS 3–4 subgroups. **A** The classification performance of the AIS and BI-RADS lexicon within the BI-RADS 3–4 subgroups. **B** The sunburst plot of the combined results in the validation set, testing set 1, and testing set 2. The inner ring represents the breast distribution of the BI-RADS 3, 4A, 4B, and 4C. Data in parentheses are a number of cases. The second ring represents the pathology distribution; dark blue means benign cases, and dark red means malignant cases. The third ring represents the prediction distribution from the AIS; blue means benign prediction, and red means malignant prediction. For example, 1439 of the breasts were BI-RADS 4B, in which 449 were benign cases and 990 were malignant, proven by pathology. The AIS can correctly classify 369 out of 449 benign cases into the benign group. For these 369 cases, the AIS can benefit them and avoid over-treatment through biopsy. In the meantime, 146 cases out of 990 malignant cases were misclassified into the benign group. Overall, the benefit cases are more than the missed cases. BI-RADS, breast imaging reporting and data system; ROC, receiver operating characteristic; AIS, artificial intelligence system

Based on the pathological results, BI-RADS categorization itself achieved accuracy of 73.15–84.09%, sensitivity of 85.51–92.32%, specificity of 58.80–74.28%, and F1 of 0.716–0.898, while AIS yielded accuracy of 85.06–96.82%, sensitivity of 76.64%–96.99%, specificity of 90.55–96.30%, and F1 of 0.802–0.979 in the validation and testing sets, respectively (Fig. 4A and Table 3). The AUC values for AIS were significantly higher in the validation set (0.828 vs 0.988, *p* < 0.001), testing set 1 (0.843 vs 0.892, *p* < 0.001), and testing set 2 (0.873 vs 0.934, *p* < 0.001).

**Table 3:** Stratified analysis of BI-RADS 3 and 4

Type	Dataset	AUC	Sensitivity (%)	Specificity (%)	Accuracy (%)	F1
BI-RADS	Validation	0.828 (0.797, 0.859)	92.3 [613/664] (90.0, 94.2)	58.8 [127/216] (51.9, 65.4)	84.1 [740/880] (81.5, 86.5)	0.898
	Testing 1	0.843 (0.821, 0.866)	85.5 [366/428] (81.8, 88.7)	65.1 [427/656] (61.3, 68.7)	73.2 [793/1084] (70.4, 75.8)	0.716
	Testing 2	0.873 (0.858, 0.888)	89.7 [1041/1161] (87.8, 91.4)	74.3 [566/762] (71.0, 77.3)	83.6 [1607/1923] (81.8, 85.2)	0.868
AIS	Validation	0.988 (0.978, 0.997)	97.0 [644/664] (95.4, 98.2)	96.3 [208/216] (92.8, 98.4)	96.8 [852/880] (95.4, 97.9)	0.979
	Testing 1	0.892 (0.871, 0.912)	76.6 [328/428] (72.3, 80.6)	90.5 [594/656] (88.0, 92.7)	85.1 [922/1084] (82.8, 87.1)	0.802
	Testing 2	0.934 (0.923, 0.945)	84.8 [984/1161] (82.6, 86.8)	92.4 [704/762] (90.3, 94.2)	87.8 [1688/1923] (86.2, 89.2)	0.893

Numbers in brackets are the numerator/denominator, and numbers in parentheses are 95% CI

*BI-RADS* breast imaging reporting and data system, *AUC* area under the receiver operating characteristic curve, *CI* confidence interval, *AIS* artificial intelligence system

In Fig. 4B, we draw a sunburst plot to visualize the benefits of AIS on BI-RADS categorization. Among 3887 breasts in the validation and testing sets, BI-RADS categorization resulted in 6.0% [233/3887] (2.1% [83/3887] BI-RADS 3 and 3.9% [150/3887] BI-RADS 4A) of false-negatives, and 13.2% [514/3887] (11.6% [449/3887] BI-RADS 4B and 1.7% [65/3887] BI-RADS 4C) of false-positives. In contrast, when we analyzed these breasts with AIS, it correctly classified 11.0% [427/3887] of BI-RADS 4B and 4C breasts into benign groups, thus avoiding excessive surgical treatment. The AIS also correctly classified 3.2% [126/3887] of BI-RADS 3 and 4A breasts into malignant groups, thus avoiding missing the best intervention time. Furthermore, 90.6% [1830/2020] of malignant BI-RADS 4B and 4C breasts were classified as malignant to ensure necessary surgery, and 96.3% [1079/1120] of benign BI-RADS 3 and 4A breasts were classified as benign to avoid unnecessary surgery. Eventually, only 5.9% [231/3887] of all BI-RADS 3–4 breasts were mistakenly stratified compared with BI-RADS categorization.

In general, AIS appropriately assigned 83.1% [427/514] of false-positives (BI-RADS 4B and C) into the benign group, meanwhile, it assigned 54.1% [126/233] of false-negatives (BI-RADS 3 and 4A) into the malignant group. The benefits of AIS are to better prevent over- and delayed-treatments.

### Re-assessment of the BI-RADS 0 with AIS assistance

In our analysis of the BI-RADS 0 subgroup, we evaluated a total of 407 breasts in the testing sets. Upon applying the AIS to all mammograms initially categorized as BI-RADS 0, radiologists successfully identified malignant lesions in 7 out of 43 breasts that were truly malignant. The AIS demonstrated a high specificity of 96.7% (352/364) in this subgroup. However, the overall sensitivity was 16.3% (7/43), reflecting the challenges in definitively categorizing BI-RADS 0 cases. (Two representative breasts are shown in Supplementary Fig. S1).

### The results of the counterbalance-designed AIS-assisted study

**Fig. 5:**
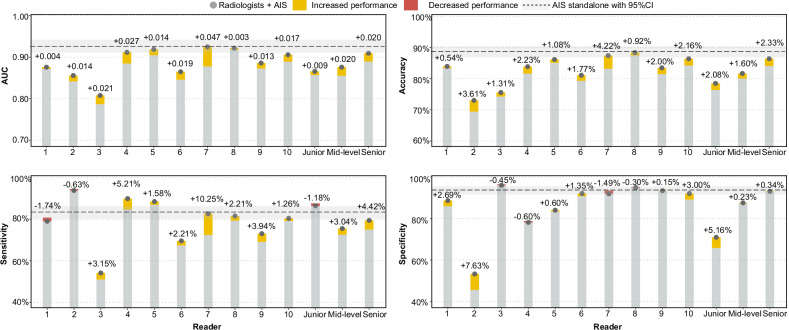
The results of the AI assistance study. Readers 1 and 2 are junior group (standardization training residents without any experience in mammography diagnosis), readers 3–6 are mid-level group (radiologists with 3–10 years of mammography diagnostic experience), and readers 7–10 are senior group (radiologists with more than 10 years of mammography diagnostic experience)

**Fig. 6:**
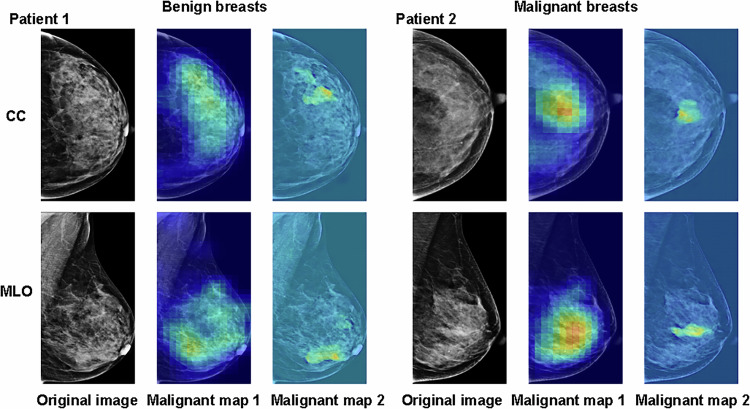
Two representative dense breasts in the AI assistance study. Patient 1 has benign breast cancer, and Patient 2 has malignant breast cancer. Patients 1 and 2 are both dense parenchyma breasts that radiologists often struggle to classify as benign or malignant. But with the assistance of AIS, most readers can tell them apart. Patient 1: mammograms for a 48-year-old woman with adenosis showed a dense gland with structural distortion in the lower outer quadrant of the left breast. With the help of AIS, the BI-RADS categories for ten readers were adjusted accordingly from 4C, 4B, 3, 4B, 4C, 3, 1, 3, 4A, 4B to 1, 4B, 3, 4B, 3, 3, 1, 3, 3, 3, respectively. Patient 2: mammograms for a 42-year-old woman with IDC showed dense gland, patchy high-density shadow, and coarse spotty calcifications in the lower inner quadrant of the left breast. With the help of AI, the BI-RADS categories for ten readers were adjusted accordingly from 3, 4B, 3, 3, 4B, 4B, 2, 4A, 3, 3 to 4C, 4C, 3, 4B, 2, 4A, 4A, 4B, 4B, 3, respectively. BI-RADS, breast imaging reporting and data system; AIS, artificial intelligence system

Among 1302 breasts assessed in the AIS-assisted study, AIS standalone achieved an AUC of 0.926 (95% CI: 0.911–0.942), sensitivity of 83.4% [529/634], specificity of 93.7% [626/668], and accuracy of 88.7% [1155/1302]. The radiologists without assistance achieved AUC = 0.787–0.919, sensitivity = 51.0% [323/634]–94.5% [599/634], and specificity = 45.7% [305/668]–96.4% [644/668], accuracy = 69.4% [904/1302]–87.5% [1139/1302], while radiologists with assistance achieved AUC = 0.808–0.925, sensitivity = 54.1% [343/634]–93.8% [595/634], and specificity = 53.3% [356/668]–96.0% [641/668], accuracy = 73.0% [951/1302]–88.4% [1151/1302]. All ten readers exhibited an increased AUC ranging from 0.003 to 0.047, where five readers exhibited significant improvements (*p* < 0.05, Fig. 5 and Supplementary Table S2). The average AUC across ten readers was significantly improved with AIS assistance (0.870 vs 0.888, *p* = 0.001). The use of AIS resulted in a trend toward increasing sensitivity in 8 out of 10 radiologists with improvements ranging from 2.2% [14/634] to 10.3% [65/634], while the average specificity slightly increased across mid-level and senior groups. Without AIS, ICC for ten readers was 0.629 (95% CI: 0.609–0.649), while with the support of AIS, ICC increased to 0.672 (95% CI: 0.653–0.690) (Table 4). We plotted two representative breasts with mass lesions (Supplementary Fig. S2) or dense parenchyma (Fig. 6) that were difficult to identify for radiologists, but with the help of AIS, most readers downgrade BI-RADS in benign cases and upgrade BI-RADS in malignant cases.

**Table 4:** The ICC results of the AI assistance study

Reader group	Reader (95% CI)	Reader + AIS (95% CI)	∆
Junior	0.337 (0.288–0.384)	0.379 (0.332–0.425)	+0.042
Mid-level	0.707 (0.686–0.727)	0.719 (0.699–0.738)	+0.012
Senior	0.682 (0.660–0.703)	0.741 (0.722–0.759)	+0.059
All	0.629 (0.609–0.649)	0.672 (0.653–0.690)	+0.043

*CI* confidence interval, *ICC* intraclass correlation coefficient

## Discussion

We developed a two-step AIS to automatically detect and diagnose breast cancer using multi-view mammograms and multi-level convolutional neural network features, and further evaluated its performance in assisting decision-making on BI-RADS categorization. We collected 24,866 breasts (49,732 digital mammograms) from Asian clinics that mainly treat symptomatic breast patients. Results showed satisfactory diagnostic performance of AIS with an AUC of 0.933–0.995 in differentiating malignant vs non-malignant breasts and 0.910–0.988 in differentiating malignant vs benign breasts in the validation and testing sets. Currently, AI for breast cancer screening has rapidly advanced from feasibility and reader studies to clinical implementation. Ezeana et al developed an iBRISK model with an AUC of 0.920–0.950, and an accuracy of 89.5% [23], which is basically consistent with our study. However, the efficiency of AI risk assessment models is subject to different AI algorithms, diverse populations, breast X-ray examination methods, and signs [24, 25].

Unlike previous large-scale AI-based mammographic studies [20, 26], that mainly established on the Caucasian population, our AIS were established among Asians, who typically have a high proportion of dense glands and blurred lesions on mammograms. However, mammography screening rate and the use of computer-aided detection (CADe) and diagnosis (CADx) are relatively low in Asia, thus, our study included both screening and diagnostic mammograms with a high proportion of accurate ground-truth labels with biopsy/surgery results.

According to the fifth edition of BI-RADS lexicon, incorrect classification between categories 3 and 4 is the main reason for high false-positive and false-negative rates in clinical practice [7, 8, 27–29]. Current criticisms of mammography focused on false positives, i.e., over-diagnosis and associated biopsies, primarily due to the wide range of predicted malignancies in the BI-RADS 4 category [28, 30]. For example, though low-risk BI-RADS 4A lesions only have a malignancy risk of 2–10%, biopsy is still required according to guidelines current BI-RADS lexicon. Due to the subjective nature of BI-RADS evaluation, clinical over-diagnosis and under-diagnosis are common events in BI-RADS category 3–4 lesions. Our AIS showed more accurate differentiation than BI-RADS categorization in categories 3–4, with significant improvements in AUC in the validation and testing sets (0.828–0.873 vs 0.892–0.988, *p* < 0.001). In addition, by correctly classifying 83.1% [427/514] of false-positive cases correctly into the benign group, and 54.1% [126/233] of false-negative cases into the malignant group, our AIS can benefit BI-RADS 3–4 categorization, perhaps avoiding unnecessary biopsies or delayed diagnosis. Comparison with prior studies, most current published works do not address the issue of the precision probability of malignancy estimation of BI-RADS category 4 mammogram suspicious findings, or address the issue of overbiopsy [21–23, 31, 32]. Because our patient data were collected from institutions that primarily treat symptomatic breast patients, the proportion of malignant cases was significantly higher than that of the screening populations. To provide balanced sensitivity and specificity and yield the highest accuracy for predicting malignancy using BI-RADS categorization, we used 4A as the threshold for malignancy, rather than 3, which could potentially affect the performance of our artificial intelligence system (AIS) and limit the generalizability of our findings to real-world clinical settings. Hence, our future studies would further validate the AIS’s performance on datasets that more closely resemble the distribution of cases seen in routine clinical practice, particularly in screening populations.

It’s crucial to understand that BI-RADS 0 is assigned when mammographic findings are indeterminate, requiring additional imaging studies for a conclusive diagnosis, such as additional mammographic imaging, ultrasound, or even MRI. Hence, our study tried to utilize AIS to identify a subset of BI-RADS 0 cases that may not require additional examinations while maintaining high specificity. In our study, AIS can successfully assist radiologists in identifying 7 out of 43 malignant lesions diagnosed with BI-RADS 0 with a specificity of 96.7% [352/364]. We acknowledge that maintaining high sensitivity is equally important to avoid missing potential malignancies. However, in the context of BI-RADS 0 cases, where further diagnostic workup is already indicated, the focus on maintaining high specificity is crucial for optimizing patient management and resource allocation. The above results strongly suggested that AIS could be an effective complement to current mammographic interpretation, especially in BI-RADS 0 and 3–4 categories [33–35].

To further investigate the potential of human-AI partnerships, we conducted a counterbalanced reader study to assess the contribution of AIS to breast imaging interpretation among radiologists across different levels. The average AUC across the ten readers was significantly improved with AIS assistance (0.870 vs 0.888, *p* = 0.001). In addition, the inter-reader agreement increased with AIS assistance. Interestingly, we found that the use of AI increased the most in the senior radiologists but the least in the junior radiologists. This may be related to the fact that the senior radiologists can identify the abnormalities suggested by AI more accurately than the junior radiologists according to their experiences [36, 37]. Currently, AI-supported mammography screening has been reported to have resulted in a similar or higher cancer detection rate compared with standard double reading [38, 39], especially for higher identifying interval and missed cancers, as well as substantially lower screen-reading workload [39], and our results are largely consistent with the above literatures [38, 39]. However, the ScreenTrustCAD trial raised concerns about the tendency of radiologists to agree over-reliance or neglect, and the study demonstrated a larger proportion of participants were recalled after initial flagging by radiologists compared with those flagged by AI CAD, with a lower proportion of cancer [40]. Hence, whether AI-supported mammography screening can replace radiologists remains to be further studied in the future.

Our study had several limitations. First, there was a significant class imbalance in our validation and testing datasets, particularly concerning the low percentage of BI-RADS 1 and 2 cases (2.7% in the validation dataset, 8.3% in the testing 1 dataset, and 7.1% in the testing 2 dataset). This imbalance is due to the fact that our patient data were collected from institutions that primarily treat symptomatic breast patients rather than screening populations. As a result, the distribution of cases in our datasets deviates from what is typically observed in clinical practice, where a vast majority of cases are classified as benign. This imbalance could potentially affect the performance of our AIS and limit the generalizability of our findings to real-world clinical settings. In a screening population, where most cases are benign, the AIS may encounter a higher number of false positives, leading to unnecessary additional imaging or biopsies. Conversely, the AIS’s performance in detecting and diagnosing malignant cases may be overestimated due to the higher prevalence of malignancies in our datasets compared to a screening population. To mitigate the impact of this limitation, future studies should validate the AIS’s performance on datasets that more closely resemble the distribution of cases seen in routine clinical practice, particularly in screening populations. Second, the mammograms in our study were captured using a single vendor (Hologic). To ensure the robustness and generalizability of our AIS, further studies should examine its performance on mammography devices from diverse vendors. Third, the effect of AIS-assisted radiological diagnosis was established using only mammograms without any clinical information, despite pathological results and follow-ups as ground truth. To better understand the potential impact of AIS in real-world clinical settings, further prospective AIS assistance studies should be conducted with and without clinical variables. Fourth, our AIS was developed using data from an Asian population. While this is a strength of our study, as it addresses the lack of research on AI-based mammography in Asian populations, it also raises the question of whether our AIS can be generalized to other populations. These findings emphasize the need for further investigation into how these higher malignancy rates might impact the performance of our model and its applicability in different clinical settings. Future studies should validate the performance of our AIS in diverse populations to assess its broader applicability.

In conclusion, AIS can serve as a clinically applicable tool to identify malignancy on mammography and further assist decision-making on the current BI-RADS lexicon, especially for stratified assessment within BI-RADS 3–4 categories to prevent over and delayed diagnosis. However, in order to assess the clinical applicability of our AIS, further studies validated in diverse populations and multiple institutions are needed in the future.

## Supplementary information

ELECTRONIC SUPPLEMENTARY MATERIAL

## Data Availability

The datasets used in this study will be available on reasonable request through contacting the corresponding author. https://github.com/QingxiaWu21/BreastMG

## Abbreviations

AIS: Artificial intelligence system
AUC: Area under the receiver operating characteristic curve
BI-RADS: Breast imaging reporting and data system
CI: Confidence interval
ICC: Intraclass correlation coefficient
ROC: Receiver operating characteristic

## References

[1] Sung H, Ferlay J, Siegel RL et al (2021) Global cancer statistics 2020: GLOBOCAN estimates of incidence and mortality worldwide for 36 cancers in 185 countries. CA Cancer J Clin 71:209–249 33538338 10.3322/caac.21660

[2] Myers ER, Moorman P, Gierisch JM et al (2015) Benefits and harms of breast cancer screening: a systematic review. JAMA 314:1615–1634 26501537 10.1001/jama.2015.13183

[3] Løberg M, Lousdal ML, Bretthauer M, Kalager M (2015) Benefits and harms of mammography screening. Breast Cancer Res Bcr 17:63 25928287 10.1186/s13058-015-0525-z PMC4415291

[4] Tabár L, Vitak B, Chen HT, Yen M, Duffy SW, Smith RA (2001) Beyond randomized controlled trials: organized mammographic screening substantially reduces breast carcinoma mortality. Cancer 91:1724–1731 11335897 10.1002/1097-0142(20010501)91:9<1724::aid-cncr1190>3.0.co;2-v

[5] Rao AA, Feneis J, Lalonde C, Ojeda-Fournier H (2016) A pictorial review of changes in the BI-RADS fifth edition. Radiographics 36:623–639 27082663 10.1148/rg.2016150178

[6] Spak DA, Plaxco JS, Santiago L, Dryden MJ, Dogan BE (2017) BI-RADS® fifth edition: a summary of changes. Diagn Interv Imag 98:179–190 10.1016/j.diii.2017.01.001 28131457

[7] Nelson HD, O’Meara ES, Kerlikowske K, Balch S, Miglioretti D (2016) Factors associated with rates of false-positive and false-negative results from digital mammography screening: an analysis of registry data. Ann Intern Med 164:226 26756902 10.7326/M15-0971 PMC5091936

[8] Huynh PT, Jarolimek AM, Daye S (1998) The false-negative mammogram. Radiographics 18:1137–1154 9747612 10.1148/radiographics.18.5.9747612

[9] Batchu S, Liu F, Amireh A, Waller J, Umair M (2021) A review of applications of machine learning in mammography and future challenges. Oncology 99:483–490 34023831 10.1159/000515698

[10] Ong M-S, Mandl KD (2017) National expenditure for false-positive mammograms and breast cancer overdiagnoses estimated at $4 billion a year. Health Affair 34:576–583 10.1377/hlthaff.2014.1087 25847639

[11] He T, Puppala M, Ezeana CF et al (2019) A deep learning-based decision support tool for precision risk assessment of breast cancer. JCO Clin Cancer Inform 3:1–12 10.1200/CCI.18.00121 PMC10445790 31141423

[12] Majid AS, de Paredes ES, Doherty RD, Sharma NR, Salvador X (2003) Missed breast carcinoma: pitfalls and pearls. Radiographics 23:881–895 12853663 10.1148/rg.234025083

[13] Yala A, Schuster T, Miles R, Barzilay R, Lehman C (2019) A deep learning model to triage screening mammograms: a simulation study. Radiology 293:38–46 31385754 10.1148/radiol.2019182908

[14] Zhu X, Wolfgruber TK, Leong L et al (2021) Deep learning predicts interval and screening-detected cancer from screening mammograms: a case-case-control study in 6369 women. Radiology 301:550–558 34491131 10.1148/radiol.2021203758 PMC8630596

[15] Yala A, Mikhael PG, Strand F et al (2021) Toward robust mammography-based models for breast cancer risk. Sci Transl Med 13:eaba4373 33504648 10.1126/scitranslmed.aba4373

[16] Aboutalib SS, Mohamed AA, Berg WA, Zuley ML, Sumkin JH, Wu S (2018) Deep learning to distinguish recalled but benign mammography images in breast cancer screening. Clin Cancer Res 24:5902–5909 30309858 10.1158/1078-0432.CCR-18-1115 PMC6297117

[17] Yala A, Lehman C, Schuster T, Portnoi T, Barzilay R (2019) A deep learning mammography-based model for improved breast cancer risk prediction. Radiology 292:60–66. 10.1148/radiol.2019182716 31063083 10.1148/radiol.2019182716

[18] Pacilè S, Lopez J, Chone P, Bertinotti T, Grouin JM, Fillard P (2020) Improving breast cancer detection accuracy of mammography with the concurrent use of an artificial intelligence tool. Radiol Artif Intell 2:e190208 33937844 10.1148/ryai.2020190208 PMC8082372

[19] Kim H-E, Kim HH, Han B-K et al (2020) Changes in cancer detection and false-positive recall in mammography using artificial intelligence: a retrospective, multireader study. Lancet Digit Health 2:e138–e148 33334578 10.1016/S2589-7500(20)30003-0

[20] McKinney SM, Sieniek M, Godbole V et al (2020) International evaluation of an AI system for breast cancer screening. Nature 577:89–94 31894144 10.1038/s41586-019-1799-6

[21] Shoshan Y, Bakalo R, Gilboa-Solomon F et al (2022) Artificial intelligence for reducing workload in breast cancer screening with digital breast tomosynthesis. Radiology 303:69–77 10.1148/radiol.211105 35040677

[22] Lotter W, Diab AR, Haslam B et al (2021) Robust breast cancer detection in mammography and digital breast tomosynthesis using an annotation-efficient deep learning approach. Nat Med 27:244–249 33432172 10.1038/s41591-020-01174-9 PMC9426656

[23] Ezeana CF, He T, Patel TA et al (2023) A deep learning decision support tool to improve risk stratification and reduce unnecessary biopsies in BI-RADS 4 mammograms. Radiol Artif Intell 5:e220259 38074778 10.1148/ryai.220259 PMC10698614

[24] Yala A, Mikhael PG, Strand F et al (2022) Multi-institutional validation of a mammography-based breast cancer risk model. J Clin Oncol 40:1732–1740 34767469 10.1200/JCO.21.01337 PMC9148689

[25] Lamb LR, Lehman CD, Gastounioti A, Conant EF, Bahl M (2022) Artificial intelligence (AI) for screening mammography, from the AJR special series on AI applications. AJR Am J Roentgenol 219:369–380 35018795 10.2214/AJR.21.27071

[26] Schaffter T, Buist DSM, Lee CI et al (2020) Evaluation of combined artificial intelligence and radiologist assessment to interpret screening mammograms. JAMA Netw Open 3:e200265 32119094 10.1001/jamanetworkopen.2020.0265 PMC7052735

[27] Lee KA, Talati N, Oudsema R, Steinberger S, Margolies LR (2018) BI-RADS 3: current and future use of probably benign. Curr Radiol Rep 6:5 29399419 10.1007/s40134-018-0266-8 PMC5787219

[28] Flowers CI, O’Donoghue C, Moore D et al (2013) Reducing false-positive biopsies: a pilot study to reduce benign biopsy rates for BI-RADS 4A/B assessments through testing risk stratification and new thresholds for intervention. Breast Cancer Res Treat 139:769–777 23764994 10.1007/s10549-013-2576-0 PMC3695318

[29] Lehman CD, Arao RF, Sprague BL et al (2017) National performance benchmarks for modern screening digital mammography: update from the breast cancer surveillance consortium. Radiology. 283:49–58 27918707 10.1148/radiol.2016161174 PMC5375631

[30] Hubbard RA, Kerlikowske K, Flowers CI, Yankaskas BC, Zhu W, Miglioretti DL (2011) Cumulative probability of false-positive recall or biopsy recommendation after 10 years of screening mammography: a cohort study. Ann Intern Med 155:481 22007042 10.1059/0003-4819-155-8-201110180-00004 PMC3209800

[31] Tari DU, Santonastaso R, De Lucia DR, Santarsiere M, Pinto F (2023) Breast density evaluation according to BI-RADS 5th edition on digital breast tomosynthesis: AI automated assessment versus human visual assessment. J Pers Med 13:609 37108994 10.3390/jpm13040609 PMC10146726

[32] Yoon J, Lee HS, Kim MJ, Park VY, Kim EK, Yoon JH (2022) AI-CAD for differentiating lesions presenting as calcifications only on mammography: outcome analysis incorporating the ACR BI-RADS descriptors for calcifications. Eur Radiol 32:6565–6574 35748900 10.1007/s00330-022-08961-7

[33] Melnikow J, Fenton JJ, Whitlock EP et al (2016) Supplemental screening for breast cancer in women with dense breasts: a systematic review for the U.S. preventive services task force. Ann Intern Med 164:268 26757021 10.7326/M15-1789 PMC5100826

[34] Yi C, Tang Y, Ouyang R et al (2022) The added value of an artificial intelligence system in assisting radiologists on indeterminate BI-RADS 0 mammograms. Eur Radiol 32:1528–1537 34528107 10.1007/s00330-021-08275-0

[35] Basha MAA, Safwat HK, Eldin AMA, Dawoud HA, Hassanin AM (2020) The added value of digital breast tomosynthesis in improving diagnostic performance of BI-RADS categorization of mammographically indeterminate breast lesions. Insights Imaging 11:26 32060736 10.1186/s13244-020-0835-2 PMC7021879

[36] Rodriguez-Ruiz A, Lång K, Gubern-Merida A et al (2019) Stand-alone artificial intelligence for breast cancer detection in mammography: comparison with 101 radiologists. J Natl Cancer Inst 111:916–922 30834436 10.1093/jnci/djy222 PMC6748773

[37] Dratsch T, Chen X, Rezazade Mehrizi M et al (2023) Automation bias in mammography: the impact of artificial intelligence BI-RADS suggestions on reader performance. Radiology. 307:e222176 37129490 10.1148/radiol.222176

[38] Lång K, Josefsson V, Larsson AM et al (2023) Artificial intelligence-supported screen reading versus standard double reading in the Mammography Screening with Artificial Intelligence trial (MASAI): a clinical safety analysis of a randomised, controlled, non-inferiority, single-blinded, screening accuracy study. Lancet Oncol 24:936–944 37541274 10.1016/S1470-2045(23)00298-X

[39] Seker ME, Koyluoglu YO, Ozaydin AN et al (2024) Diagnostic capabilities of artificial intelligence as an additional reader in a breast cancer screening program. Eur Radiol 34:6145–6157 38388718 10.1007/s00330-024-10661-3 PMC11364680

[40] Dembrower KE, Crippa A, Eklund M, Strand F (2025) Human-AI interaction in the screenTrustCAD trial: recall proportion and positive predictive value related to screening mammograms flagged by AI CAD versus a human reader. Radiology 314:e242566 40100021 10.1148/radiol.242566

