# Supplementary material for: Mammography-based artificial intelligence for breast cancer detection, diagnosis, and BI-RADS categorization using multi-view and multi-level convolutional neural networks
Source: Insights Imaging. 2025 May 21;16:109. doi: 10.1186/s13244-025-01983-x (PMC12095762; doi:10.1186/s13244-025-01983-x)
Supplement: Supplementary file 1 — ELECTRONIC SUPPLEMENTARY MATERIAL [file 13244_2025_1983_MOESM1_ESM.pdf]

Mammography-based Artificial Intelligence System for Breast Cancer Detection, Diagnosis, and BI-RADS Categorization Using Multi-View and Multi-Level Convolutional Neural Networks

ELECTRONIC SUPPLEMENTARY MATERIAL

Supplementary Method 1. Development of AIS.

In this study, a two-step breast cancer classification method was proposed to fully use local lesion information and macroscopic malignancy information (Fig. 2Aand Fig. 2B). 1) A patch-level multi-task network was proposed to jointly learn discriminative lesion localization and lesion characteristics (malignant or non-malignant). Raw images were randomly cropped into 512×512 patches and fed into the multi-task network for patch-level tumor segmentation and classification. We used 2000 annotated mammograms to train the patch-level model. Note that patch-level annotations were necessary for the networks. 2) Based on the patch-level outputs, two are merged into image-level maps, one from classification results (Malignant Map 1) and the other from segmentation results (Malignant Map2). Then, we combined the original image and these two maps as a three-channel input to train an EfficientNet-B0 for image-level classification, aiming to learn the macroscopic malignancy features. The network outputs for craniocaudal (CC) and mediolateral oblique (MLO) views were then averaged to get the breast-level cancer classification for each breast. In this stage, the training required no annotations but image-level malignant labels. The breast-level model was trained with additional 22598 mammograms (11299 breasts) without patch-level annotation, and evaluated in the internal validation set consisting of 5648 mammograms (2824 breasts) and independent external testing sets consisting of 19486 mammograms (9743 breasts).”

Supplementary Method 2. The interface of AI assistance study.

On the AI-aided interface, experts can see a red contour of the suspicious area and probability of malignancy predicted by the AIS. Experts had access to the suspicious area immediately when loading mammograms, and the suspicious area could be toggled off to reveal the unaltered mammograms. Given the AIS results, experts had the option to take it into consideration or disregard it based on clinical judgment."

Table S1 The clinical characteristics and mammographic assessments.

Dataset	Training	Validation	Testing 1	Testing 2
No.	4320	1051	1516	2453
Pathology (%)				
Malignant	3237 (74.9)	776 (73.8)	529 (34.9)	1366 (55.7)
Benign	1083 (25.1)	275 (26.2)	987 (65.1)	1087 (44.3)
BI-RADS (%)				
0	131 (3.0)	39 (3.7)	219 (14.4)	188 (7.7)
1	18 (0.4)	1 (0.1)	18 (1.2)	37 (1.5)
2	101 (2.3)	27 (2.6)	108 (7.1)	138 (5.6)
3	391 (9.1)	99 (9.4)	352 (23.2)	424 (17.3)
4A	340 (7.9)	79 (7.5)	137 (9.0)	262 (10.7)
4B	1442 (33.4)	358 (34.1)	348 (23.0)	733 (29.9)
4C	1412 (32.7)	344 (32.7)	247 (16.3)	504 (20.5)
5	485 (11.2)	104 (9.9)	87 (5.7)	167 (6.8)

Note.— Data are the total number of breasts with definitive pathological results (malignant or benign) and had consistent evaluation from two senior radiologists, and are used for subgroup analysis. Thus the number of samples in Table S1 is smaller than those in Table 1. BI-RADS = Breast Imaging Reporting and Data System.

Table S2 The results of AI assistance study.

AUC				
**Reader**	**reader**	**reader + AIS**	**Δ**	***P***
**1**	0.872 (0.852,0.891)	0.876 (0.857,0.895)	0.004	0.670
**2**	0.842 (0.822,0.862)	0.856 (0.837,0.876)	0.014	0.199
**3**	0.787 (0.764,0.809)	0.808 (0.787,0.829)	0.021	**0.010**
**4**	0.885 (0.867,0.903)	0.912 (0.897,0.928)	0.027	**<0.001**
**5**	0.905 (0.888,0.922)	0.919 (0.903,0.934)	0.014	0.073
**6**	0.846 (0.826,0.866)	0.865 (0.846,0.884)	0.019	**0.015**
**7**	0.878 (0.859,0.898)	0.925 (0.911,0.940)	0.047	**<0.001**
**8**	0.919 (0.903,0.934)	0.922 (0.907,0.937)	0.003	0.436
**9**	0.873 (0.854,0.892)	0.886 (0.868,0.905)	0.013	0.181
**10**	0.889 (0.871,0.907)	0.906 (0.889,0.923)	0.017	**0.014**
**Sensitivity (%)**				
**1**	80.8 [512/634] (77.5,83.8)	79.0 [501/634] (75.6,82.1)	-1.7 [11/634]	0.305
**2**	94.5 [599/634] (92.4,96.1)	93.8 [595/634] (91.7,95.6)	-0.6 [4/634]	0.537
**3**	51.0 [323/634] (47.0,54.9)	54.1 [343/634] (50.1,58.0)	3.2 [20/634]	**0.022**
**4**	84.7 [537/634] (81.7,87.4)	89.9 [570/634] (87.3,92.1)	5.2 [33/634]	**<0.001**
**5**	86.9 [551/634] (84.0,89.4)	88.5 [561/634] (85.7,90.9)	1.6 [10/634]	0.189
**6**	67.3 [427/634] (63.5,71.0)	69.6 [441/634] (65.8,73.1)	2.2 [14/634]	0.170
**7**	72.4 [459/634] (68.7,75.8)	82.7 [524/634] (79.5,85.5)	10.3 [65/634]	**<0.001**
**8**	79.3 [503/634] (76.0,82.4)	81.5 [517/634] (78.3,84.5)	2.2 [14/634]	**0.043**
**9**	69.1 [438/634] (65.3,72.7)	73.0 [463/634] (69.4,76.5)	3.9 [25/634]	**0.017**
**10**	79.2 [502/634] (75.8,82.3)	80.4 [510/634] (77.1,83.5)	1.3 [8/634]	0.371
**Specificity (%)**				
**1**	85.9 [574/668] (83.1,88.5)	88.6 [592/668] (86.0,90.9)	2.7 [18/668]	0.080
**2**	45.7 [305/668] (41.8,49.5)	53.3 [356/668] (49.4,57.1)	7.6 [51/668]	**0.002**
**3**	96.4 [644/668] (94.7,97.7)	96.0 [641/668] (94.2,97.3)	-0.4 [3/668]	0.513
**4**	78.7 [526/668] (75.4,81.8)	78.1 [522/668] (74.8,81.2)	-0.6 [4/668]	0.719
**5**	83.4 [557/668] (80.3,86.1)	84.0 [561/668] (81.0,86.7)	0.6 [4/668]	0.705
**6**	90.7 [606/668] (88.3,92.8)	92.1 [615/668] (89.8,94.0)	1.3 [9/668]	0.233
**7**	93.4 [624/668] (91.3,95.2)	91.9 [614/668] (89.6,93.9)	-1.5 [10/668]	0.132
**8**	95.2 [636/668] (93.3,96.7)	94.9 [634/668] (93.0,96.5)	-0.3 [2/668]	0.637
**9**	93.3 [623/668] (91.1,95.0)	93.4 [624/668] (91.3,95.2)	0.1 [1/668]	0.893
**10**	89.1 [595/668] (86.5,91.3)	92.1 [615/668] (89.8,94.0)	3.0 [20/668]	**0.017**
**Accuracy (%)**				
**1**	83.4 [1086/1302] (81.3,85.4)	84.0 [1093/1302] (81.8,85.9)	0.5 [7/1302]	0.750
**2**	69.4 [904/1302] (66.8,71.9)	73.0 [951/1302] (70.5,75.4)	3.6 [47/1302]	**0.046**

**3**	74.3 [967/1302] (71.8,76.6)	75.6 [984/1302] (73.2,77.9)	1.3 [17/1302]	0.469
**4**	81.6 [1063/1302] (79.4,83.7)	83.9 [1092/1302] (81.8,85.8)	2.2 [29/1302]	0.146
**5**	85.1 [1108/1302] (83.0,87.0)	86.2 [1122/1302] (84.2,88.0)	1.1 [14/1302]	0.468
**6**	79.3 [1033/1302] (77.0,81.5)	81.1 [1056/1302] (78.9,83.2)	1.8 [23/1302]	0.279
**7**	83.2 [1083/1302] (81.0,85.2)	87.4 [1138/1302] (85.5,89.2)	4.2 [55/1302]	**0.003**
**8**	87.5 [1139/1302] (85.6,89.2)	88.4 [1151/1302] (86.5,90.1)	0.9 [12/1302]	0.508
**9**	81.5 [1061/1302] (79.3,83.6)	83.5 [1087/1302] (81.4,85.5)	2.0 [26/1302]	0.197
**10**	84.2 [1097/1302] (82.2,86.2)	86.4 [1125/1302] (84.4,88.2)	2.2 [28/1302]	0.135

Note.— Numbers in brackets are numerator/denominator, and numbers in parentheses are 95%CI. AUC = area under the receiver operating characteristic curve. AIS = artificial intelligence system.

Supplementary Figures and Legends

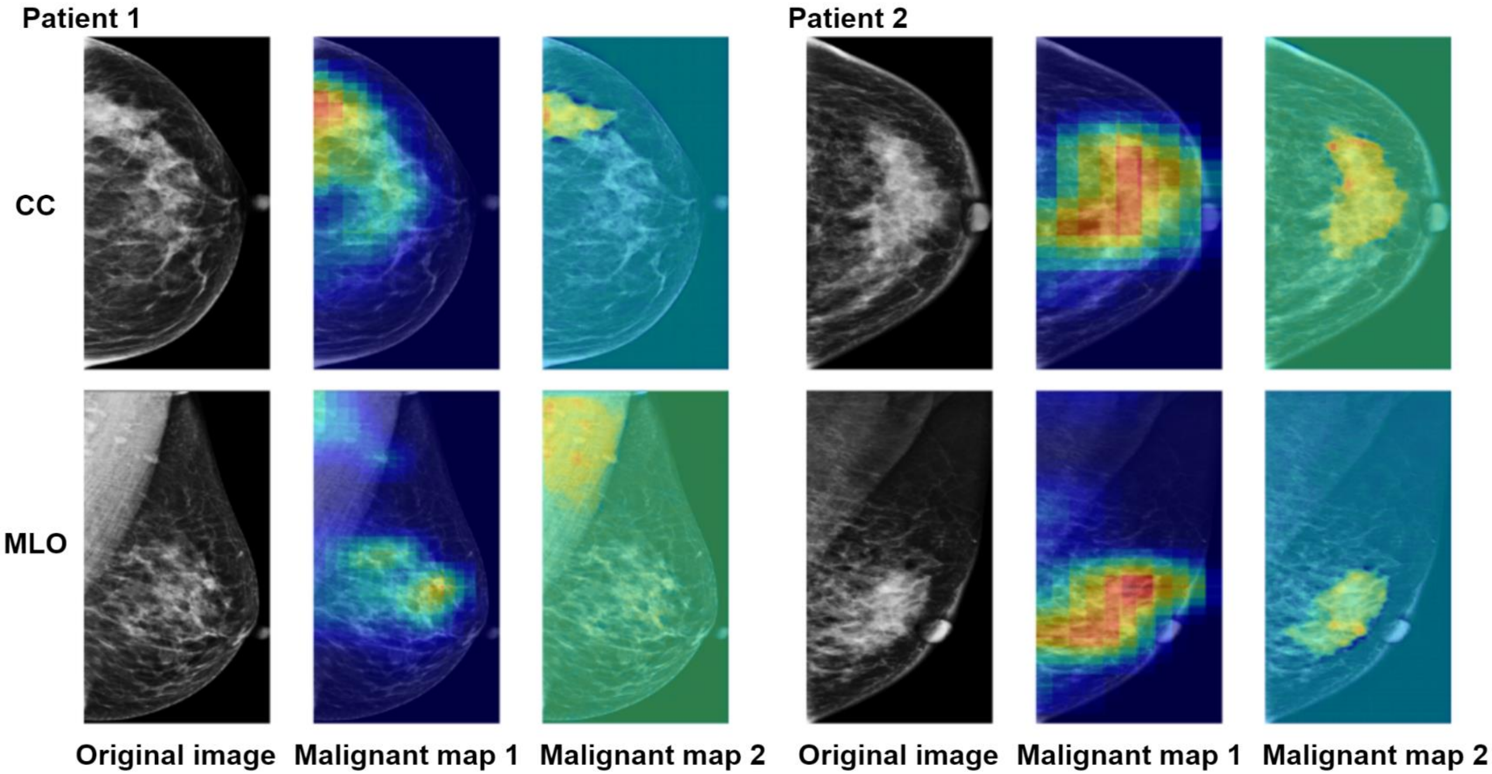

Figure S1. Two representative malignant breasts from the BI-RADS 0 in the testing set.

Patient 1 and 2 were initially diagnosed with BI-RADS 0, and further succeeded to be found by radiologists when re-evaluating with the support of AIS. Patient 1: Mammograms for a 43-year-old woman with DCIS in the left breast was classified as BI-RADS 0 category because only a suspected patchy density was found on the outer quadrant of CC view. The malignant lesion was succeeded to be found through the heatmap of malignant lesion characteristics in the corresponding region both on Malignant Map 1 and Malignant Map 2. Patient 2: Mammograms for a 44-year-old woman with IDC in the left breast was classified as BI-RADS 0 category because no abnormality was found except for structural disorder and dense gland on mammograms. The malignant lesion was succeeded to be found through the heatmap of malignant lesion characteristics both on Malignant Map 1 and Malignant Map 2, which was confirmed intraoperatively an about 5cm*6cm mass locating at 12 o'clock nearby the nipple in the left breast. AIS = artificial intelligence system.

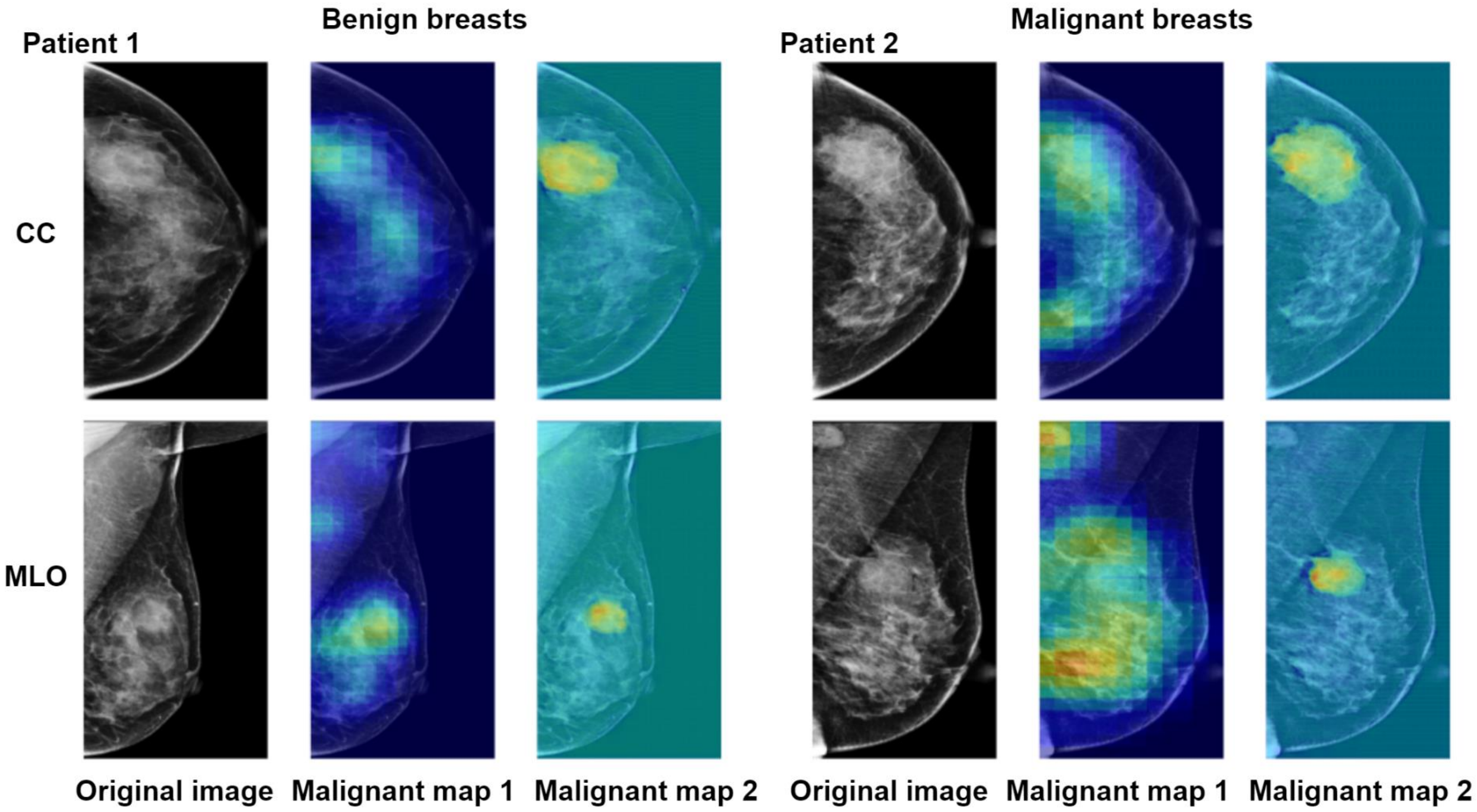

Figure S2. Two representative breasts with mass lesions in the AI assistance study. Patient 1 is benign breast, and Patient 2 and 4 are malignant breast. Patient 1 and 2 are both soft tissue lesion, and usually very difficult for radiologists to classify them into benign or malignant group. But with the assistance of our AIS, most readers can discriminate them. Patient 1: Mammograms for a 52-year-old woman with fibroadenoma showed an ill-defined mass in the outer upper quadrant of right breast. With AI assistance, the BI-RADS categories for 10 readers were adjusted accordingly from 4C, 4B, 4B, 4C, 4B, 4B, 1, 3, 4B, 4C to 3, 4A, 3, 4B, 4A, 4A, 1, 3, 3, 4B, respectively.

Patient 2: Mammograms for a 43-year-old woman with IDC showed an ill-defined mass in the outer upper quadrant of right breast. With AI assistance, the BI-RADS categories for 10 readers were adjusted accordingly from 2, 4C, 3, 4B, 4B, 4B, 4B, 4A, 3, 4C to 4C, 4C, 4A, 4B, 4B, 4B, 4C, 4A, 4A, 4C, respectively.
